# Affective lability and social functioning in severe mental disorders

**DOI:** 10.1007/s00406-022-01380-1

**Published:** 2022-01-27

**Authors:** Margrethe Collier Høegh, Ingrid Melle, Sofie R. Aminoff, Stine Holmstul Olsen, Synve Hoffart Lunding, Torill Ueland, Trine Vik Lagerberg

**Affiliations:** 1 NORMENT, Centre for Research On Mental Disorders, Division of Mental Health and Addiction, Institute of Clinical Medicine, University of Oslo and Oslo University Hospital, Bygg 49, Ullevål sykehus, Nydalen, PO Box 4956, 0424 Oslo, Norway; 2 Division of Mental Health and Addiction, Oslo University Hospital, Oslo, Norway; 3 Department of Psychology, University of Oslo, Oslo, Norway

**Keywords:** Affective lability, Social functioning, Psychotic disorders, Schizophrenia spectrum, Bipolar spectrum, Affective lability Scale Short Form (ALS-SF)

## Abstract

Social functioning is impaired in severe mental disorders despite clinical remission, illustrating the need to identify other mechanisms that hinder psychosocial recovery. Affective lability is elevated and associated with an increased clinical burden in psychosis spectrum disorders. We aimed to investigate putative associations between affective lability and social functioning in 293 participants with severe mental disorders (schizophrenia- and bipolar spectrum), and if such an association was independent of well-established predictors of social impairments. The Affective Lability Scale (ALS-SF) was used to measure affective lability covering the dimensions of anxiety-depression, depression-elation and anger. The interpersonal domain of the Social Functioning Scale (SFS) was used to measure social functioning. Correlation analyses were conducted to investigate associations between affective lability and social functioning, followed by a hierarchical multiple regression and follow-up analyses in diagnostic subgroups. Features related to premorbid and clinical characteristics were entered as independent variables together with the ALS-SF scores. We found that higher scores on all ALS-SF subdimensions were significantly associated with lower social functioning (*p* < 0.005) in the total sample. For the anxiety-depression dimension of the ALS-SF, this association persisted after controlling for potential confounders such as premorbid social functioning, duration of untreated illness and current symptoms (*p* = 0.019). Our results indicate that elevated affective lability may have a negative impact on social functioning in severe mental disorders, which warrants further investigation. Clinically, it might be fruitful to target affective lability in severe mental disorders to improve psychosocial outcomes.

**Supplementary Information:**

The online version contains supplementary material available at 10.1007/s00406-022-01380-1.

## Introduction

Social functioning, defined as the capacity of a person to function in different societal roles such as homemaker, worker, student, partner, family member or friend [1, 2], is an important marker of recovery and a predictor of quality of life in severe mental disorders [3, 4]. Social impairments are present across schizophrenia- and bipolar spectrum disorders and appear to be driven by a range of factors. A better understanding of the different paths leading to social impairment is important to tailor and personalize interventions for the individual patient. Affective disturbances, defined broadly as disruptions in the subjective experience, expressive behavior and physiology of emotions and mood [5], are taxing and highly prioritized as treatment targets by the patients [6–8]. Several studies have found significant associations between various forms of dysregulated affect and reduced social functioning in patients with both non-affective and affective psychotic disorders [9–15]. This association appears to be independent of other risk factors such as neurocognitive- and social cognitive deficits, indicating that affective dysregulation may uniquely contribute to social impairments in psychosis. As human emotions are developed, expressed and regulated in interaction with others, it is perhaps not surprising that challenges with affect regulation make social contexts and situations particularly burdensome [16]. Still, there is a paucity of studies investigating the role of specific  facets of affective dysregulation for social functioning in severe mental disorders [17, 18]. Affective lability refers to the propensity to experience rapid, excessive and unpredictable changes in affective states and is associated with poor clinical and functional outcome in many psychiatric disorders [19, 20]. In a sample partially overlapping with that of the current study, we have previously found that affective lability is elevated in schizophrenia- and bipolar spectrum disorders compared to healthy controls [21]; with the highest level in bipolar II disorder (BDII) and equally high levels in schizophrenia and bipolar I disorder (BDI) [22]. Hence, affective lability appears to be a common illness feature across these disorders, with potential consequences for clinical outcome.

To clarify the relationship between affective lability and social functioning in severe mental disorders, other known risk factors for social impairment must be taken into consideration. Predictors of social impairment appear similar across the disorders, and range from individual characteristics through lifetime- and current illness-related features [23, 24]. As social impairment is higher in schizophrenia compared to schizoaffective- and bipolar disorders [23], the presence and/or prominence of psychotic symptoms may be of relevance. This is supported by findings of larger functional impairment in patients with bipolar disorder with psychotic symptoms compared to those without [25–27]. Nonetheless, the severity of affective symptoms, depressive in particular, also seems to predict social functioning across diagnoses [28–33]. Hence, core clinical symptoms, both current and over the lifetime, appear to be central to social functioning in these populations. In addition, there are several other shared risk factors for social impairments highlighted in the literature. These include male sex [34, 35], poor premorbid social functioning [36, 37], neurocognitive deficits [25, 38], total number of illness episodes [28, 39], duration of untreated illness [40, 41], negative symptoms including apathy [23, 33, 42] and comorbidity such as substance use and anxiety [28, 43–46].

Here, we aim to investigate the relationship between affective lability and social functioning in severe mental disorders, and to explore whether this putative relationship is specific to subdimensions of affective lability. To our knowledge, this relationship has not been investigated previously. We hypothesize that affective lability will be associated with social functioning independent of other pre-defined predictors of social impairment across severe mental disorders.

## Methods

### Participants

The study sample was comprised of two hundred and ninety-three participants with severe mental disorders (schizophrenia [*n* = 62]; schizophreniform [*n* = 13]; schizoaffective [*n* = 16]; BDI [*n* = 102]; BDII [*n* = 68]; psychosis Not Otherwise Specified (NOS) [*n* = 32]), recruited through the Thematically Organized Psychosis (TOP) research study at the Norwegian Center for Mental Disorders Research (NORMENT) in Oslo, Norway. Recruitment to the TOP study is consecutive and still ongoing via psychiatric inpatient and outpatient units in a catchment area that is comprised of all the major hospitals in Oslo. All participants in the study must meet diagnostic criteria for a Diagnostic and Statistical Manual of Mental Disorders 4^th^ Edition (DSM-IV) diagnosis of schizophrenia- or bipolar spectrum disorders and be able to give informed consent. In addition, exclusion criteria are intelligence quotient (IQ) below 70, prior history of severe head trauma and insufficient understanding of a Scandinavian language. In the current study, only participants who had completed the Affective Lability Scale—Short Form (ALS-SF) and the Social Functioning Scale (SFS) were included.

The TOP study has been approved by the Regional Committee for Medical Research Ethics and the Norwegian Data Inspectorate and is conducted in line with the Helsinki declaration of 1975 (as revised in 2008 and 2013).

### Diagnostic assessment

The Structured Clinical Interview for DSM-IV axis 1 disorders (SCID; modules A-E) [47] was used to establish diagnoses in the study as part of a thorough clinical assessment carried out by clinical psychologists, medical doctors in psychiatric residency or psychiatrists. All clinical personnel in the study undergo an extensive 3-month training and quality assurance program in the use of SCID and the Positive and Negative Syndrome Scale (PANSS) developed at the University of California, Los Angeles, USA [48] before being allowed to carry out clinical interviews with diagnostic assessments, irrespective of previous clinical training. Diagnostic reliability across different groups of assessment teams have demonstrated a Cohen’s kappa for diagnosis in the range between 0.92 and 0.99.

### The Social Functioning Scale (SFS)

The Social Functioning Scale is a self-report scale that was originally developed to measure social adjustment in patients with schizophrenia, tapping areas of functioning that are crucial to community living [49]. It has later been validated for use with other severe mental disorders, including bipolar disorder, and has been found to have sound psychometric properties, as well as to correlate highly with clinician-rated measures of functioning [3, 24, 50–53]. The scale is comprised of 76 items that are rated on a Likert scale and yields a total score of overall functioning after illness debut, as well as scores on seven subscales: (1) social engagement/withdrawal (amount of time spent alone, likelihood of initiating conversations, social avoidance); (2) interpersonal behavior (number of friends, romantic relationships, quality of communication); (3) prosocial activities (engagement in common social activities, e.g. going to the cinema); (4) recreation (engagement in hobbies/activities); (5) independence-competence (ability to maintain independent living, e.g. shopping for groceries); (6) independence-performance (performance of skills required for independent living); (7) employment/occupation (or being a full-time student). Each subscale is standardized and normalized to a scaled score (SS) with a mean of 100 and a standard deviation of 15, and the full-scale score is calculated as the mean of the SSs of the seven subscales [49]. The first two subscales combined are referred to as the SFS interpersonal domain. This domain has been found to have good ecological validity and to capture social isolation and social avoidance in particular [54], which are in themselves risk factors for depression, loneliness and other negative health outcomes [16]. The 3rd and 4th subscales comprise the activity domain, and although it includes single items that may reflect social functioning (i.e. whether you have visited friends), it has been found to have low ecological validity [54]. The remaining three subscales are not reflective of *social* functioning per se, but rather encompass skills for independent living (budgeting, preparing a meal, etc.) and ability to work/study which were not of primary interest in this respect. Consequently, only the interpersonal domain was used for the present study as this domain best represents our outcome measure of interest, namely social functioning. A higher score on the SFS interpersonal domain is indicative of a higher level of functioning.

### The Affective Lability Scale Short Form (ALS-SF)

We used the Affective lability Scale Short Form (ALS-SF) [55] to measure affective lability. The scale, which is filled in by the participant, yields a total level of affective lability, in addition to subscores covering fluctuations between three subdimensions; anxiety-depression, depression-elation and anger-normal mood. The scale contains 18 items that are rated on a 4-point Likert scale ranging from 0 (“very uncharacteristic of me”) to 3 (“very characteristic of me”) and has been found to have good psychometric properties [21, 56, 57]. Of the items, five refer to shifts in anxiety-depression, eight refer to shifts in depression-elation and the final five items cover shifts between anger-normal mood. The ALS-SF yields subscores for the three subdimensions in addition to a total score of affective lability (the sum of all item responses divided by 18). In the current study, we chose to investigate the subdimensions in the total sample as opposed to the composite (total) ALS-SF score to more specifically address if there are certain types of affective lability that appear to be linked to social functioning.

### Potential confounders of the relationship between social functioning and affective lability

The following variables are previously established predictors of social functioning considered potential confounders of the relationship between social functioning and affective lability in the current analyses. With respect to individual characteristics we investigated: sex, premorbid social functioning based on scores on the social domain in childhood from the Premorbid Adjustment Scale (PAS) [58, 59], as well as overall cognitive ability measured by the Wechsler Abbreviated Scale of Intelligence (WASI, [60]). More specific investigations of the role of cognitive deficits on social functioning were beyond the scope of the current study. Features related to illness course included estimation of duration of illness which was based on the age of onset of the first SCID-verified episode of psychosis for schizophrenia, schizophreniform, schizoaffective and psychosis NOS, and the first SCID-verified affective episode for BDI and BDII. We also calculated an estimate for the duration of *untreated* illness. For schizophrenia, schizophreniform, schizoaffective and psychosis NOS, duration of untreated psychosis (DUP) was calculated as the number of weeks from the first SCID-verified psychotic episode to adequate treatment (antipsychotic medication in adequate doses/admission to hospital for psychosis). For BDI and BDII, the duration of untreated bipolar disorder (DUB) was based on the number of weeks from the first SCID-verified episode of mania/hypomania to adequate treatment (mood-stabilizing medication or antipsychotics in adequate doses/hospital admission for treatment of mania). DUP and DUB were combined into one variable, duration of untreated illness, to use in the analyses of the whole sample. Further, the total number of illness episodes was calculated as the sum of all recorded illness episodes (depressive, hypomanic, manic, mixed, psychotic). Based on previous indications of a relationship between psychotic symptoms and lower social functioning and since the present sample also included individuals with bipolar disorder who have never had a psychotic episode, a categorical psychosis lifetime variable was made which denoted the lifetime history of a SCID-verified psychotic episode. With respect to current symptom states, they were assessed with the following: positive psychotic symptoms with the positive subscale of the PANSS [61], negative symptoms with the negative subscale of the PANSS, manic symptoms with the Young Mania Rating Scale (YMRS [62]), and depressive symptoms were assessed with the depression item (G6) in the general scale of the PANSS. To measure comorbid anxiety symptoms, the anxiety item (G2) from the general scale of the PANSS was used. These items from the general scale of the PANSS were chosen because they were the only measures of depression and anxiety collected at the same time point as the ALS-SF and the SFS for all participants. We further used the Alcohol Use Disorders Identification Test (AUDIT, [63]) and the Drug Use Disorders Identification Test (DUDIT, [64]) to measure the degree of harmful substance use since associations between reduced social functioning and substance use has previously been found [65, 66].

### Statistical analyses

**Fig. 1:**
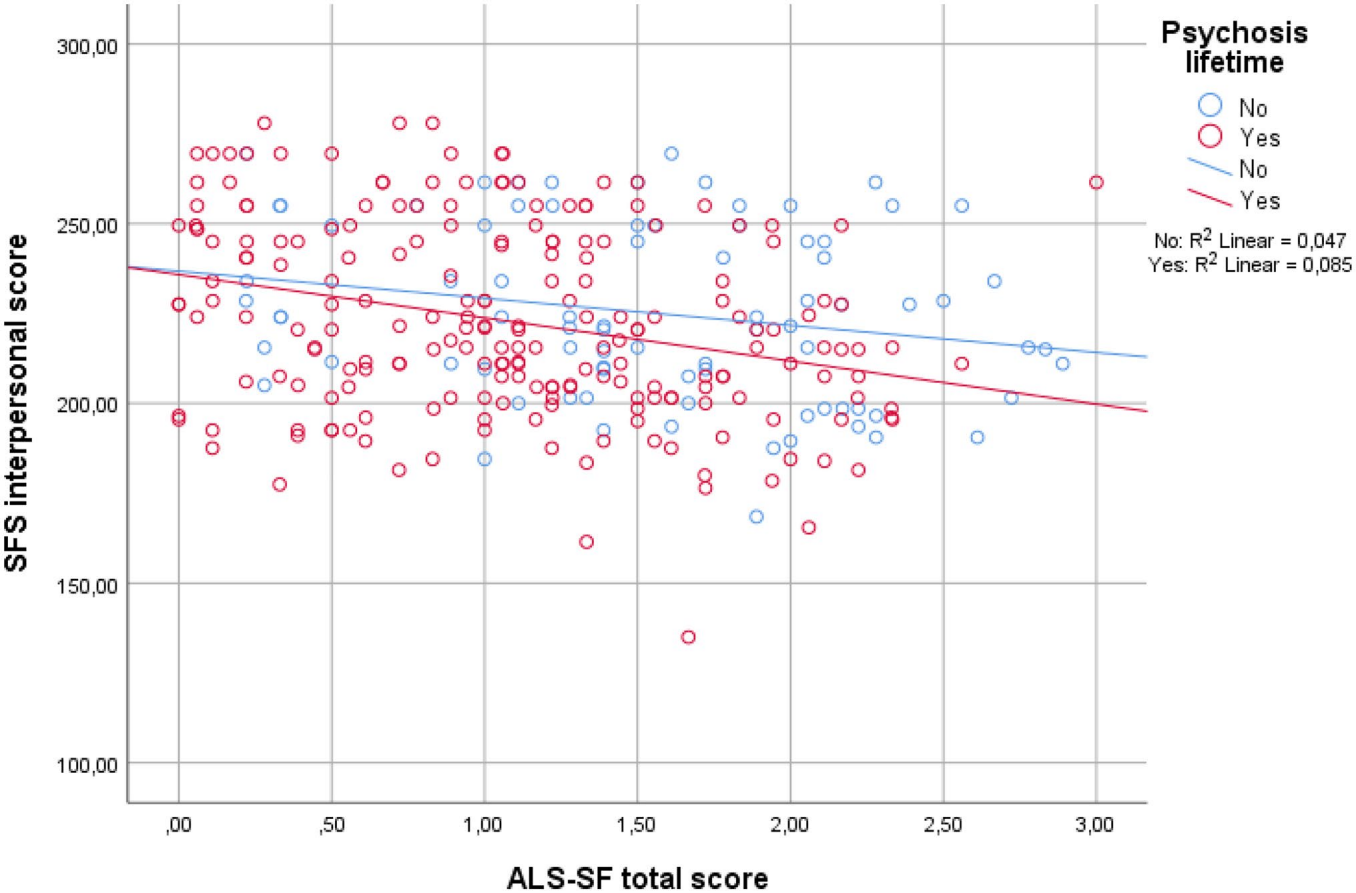
The relationship between affective lability and social functioning split by the presence of lifetime psychosis

**Fig. 2:**
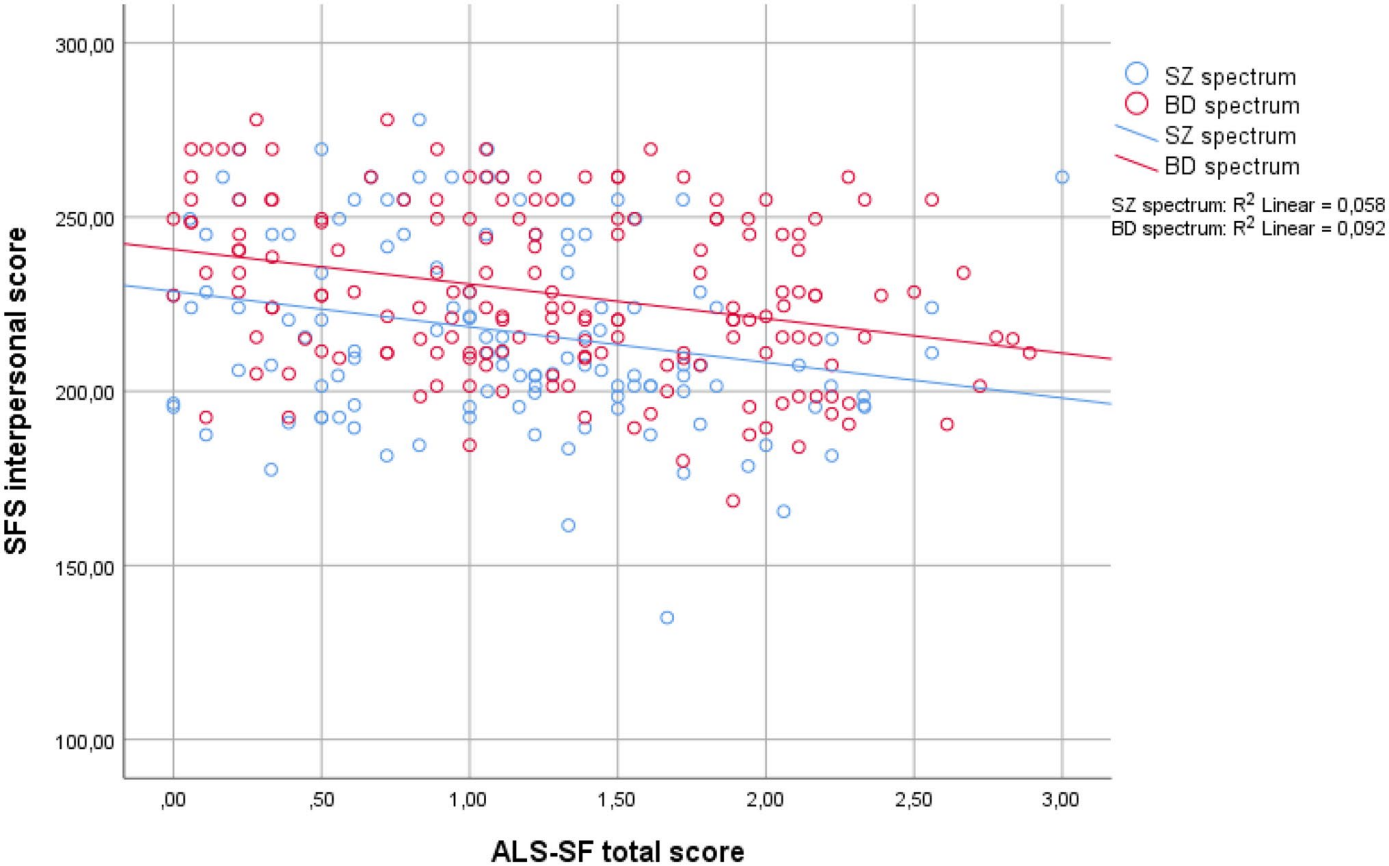
The relationship between affective lability and social functioning split by diagnostic group

Demographic and clinical characteristics of the sample were investigated with descriptive statistics, including means with standard deviations or frequencies with percentages as fitted. Pearson and Spearman’s correlations were conducted to investigate the relationship between the SFS interpersonal domain and ALS-SF dimensions. Correlational analyses were also performed to investigate the relationship between the SFS interpersonal and demographic as well as clinical variables that have been established as predictors of social functioning in previous research. This was followed by a hierarchical multiple linear regression analysis for the SFS interpersonal score entering all the variables that were significantly associated with the SFS score. The analysis was conducted block-wise to investigate the proportion of variance explained by affective lability specifically. Here, premorbid social adjustment was entered first, the illness course variables (duration of untreated illness, total number of illness episodes) next, followed by the current symptom- and comorbidity variables (positive- and negative symptoms, manic symptoms, depression and anxiety) and finally all of the ALS-SF subdimensions in the last block. There were no indications of problematic multicollinearity between the ALS-SF subdimensions (tolerance ≥ 0.35 and VIF ≤ 2.9 for all dimensions). Based on our previous findings of higher levels of affective lability in BDII versus BDI and schizophrenia [67] and lower levels of social functioning in schizophrenia and psychotic versus non-psychotic bipolar disorder, we anticipated a possible interaction effect between lifetime psychosis and affective lability on social functioning. However, visual inspections of a scatterplot of the relationship between SFS and ALS-SF split by the dichotomous psychosis lifetime variable (Fig. 1) did not indicate an interaction between ALS-SF and psychosis lifetime on social functioning. Thus, we did not include such an interaction term in the regression analysis. As there could be differences between the diagnostic groups in terms of social functioning and to further disentangle putative relationships, follow-up analyses were also carried out in diagnostic subgroups according to current diagnostic nomenclature: schizophrenia spectrum (schizophrenia, schizophreniform, schizoaffective, psychosis NOS; *n* = 123) and bipolar spectrum (BDI and BDII; *n* = 170). Here, separate bivariate analyses for the two groups were performed to investigate the association between social functioning and affective lability, in addition to the other relevant demographic and clinical variables. The variables that were significantly associated with the SFS interpersonal score in bivariate analyses for each group were then entered into separate forced entry hierarchical multiple regression models. Due to lower n when the sample was split, the total score of the ALS-SF was used in the multivariate analyses for both groups to ensure enough statistical power when all predictor variables were entered. An interaction term between affective lability and diagnostic subgroup on social functioning was not included as a scatterplot did not indicate the presence of such an interaction (Fig. 2).

All statistical analyses were performed using the Statistical Package for the Social Sciences (SPSS Inc., Chicago, IL, version 26) and a significance level of *p* ≤ 0.05 (two-tailed tests) was employed.

## Results

### Demographics and clinical characteristics of the sample

Demographic and clinical characteristics of the sample are presented in Table 1. There were 82 participants without lifetime psychosis; 25/102 (24.5%) in BDI and 57/68 (83.8%) in BDII.

**Table 1:** Demographics and clinical characteristics

	Total sample, *n* = 293	Schizophrenia-spectrum, *n* = 123	Bipolar-spectrum, *n* = 170	Statistics	*p* value
	Mean (SD)	Mean (SD)	Mean (SD)		
Age (years)	30.1 (9.9)	29.7 (8.9)	31 (10.5)	*t* = − 1.092, df = 284	0.276
Female sex, *n* (%)	157 (53.0)	53 (43.1)	101 (59.4)	*X*^2^ = 7.625, df = 2	**0.006** BD > SZ
SFS interpersonal	223.4 (25.9)	217.1 (27.4)	227.9 (24.1)	*t* = − 3.586, df = 291	**0.000** **BD > SZ**
Duration of illness, years^a^	8.6 (9.0)	4.9 (6.6)	11.2 (9.5)	*t* = − 6.675, df = 288	**0.000** BD > SZ
IQ (WASI)^b^	108 (13.4)	104 (14.8)	110.9 (11.5)	*t* = − 4.277, df = 204	**0.000** BD > SZ
Total number of illness episodes	9.4 (16.2)	3.9 (4.8)	13.4 (19.9)	*t* = − 5.981, df = 196	**0.000** BD > SZ
Onset of illness ≤ 18 years, *n* (%)	123 (42.0)	25 (20.3)	98 (57.6)	*X*^2^ = 40.813, df = 1	**0.000** BD > SZ
Duration of untreated illness, weeks^c^	47 (145.3)	75 (173.3)	22 (109.5)	*t* = 2.764, df = 226	**0.008** SZ > BD
Premorbid social functioning (PAS)^d^	1.9 (2.3)	2.1 (2.4)	1.8 (2.3)	*t* = 1.196, df = 286	0.233
Psychosis lifetime, *n* (%)	214 (72.1)	123 (100)	88 (52)	*X*^2^ = 82.386, df = 1	**0.000** SZ > BD
PANSS—total	47.8 (13.3)	55.4 (15.1)	42.3 (8.3)	*t* = 8.702, df = 175	**0.000** SZ > BD
PANSS—Positive	10.4 (3.9)	12.6 (4.4)	8.9 (2.5)	*t* = 8.488, df = 178	**0.000** SZ > BD
PANSS—Negative	11.2 (4.8)	14.0 (5.7)	9.2 (2.5)	*t* = 8.797, df = 157	**0.000** SZ > BD
Depression (PANSS item G6)	2.4 (1.3)	2.3 (1.2)	2.5 (1.4)	*t* = − 1.448, df = 291	0.149
Anxiety (PANSS item G2)	2.8 (1.3)	2.7 (1.2)	3.1 (1.4)	*t* = − 1.688, df = 291	0.092
YMRS—total^e^	2.6 (3.6)	2.7 (3.6)	2.5 (4.1)	t = .490, df = 288	0.624
AUDIT^f^	6.8 (6.0)	5.2 (4.9)	8.1 (6.5)	*t* = − 4.116, df = 282	**0.000** BD > SZ
DUDIT^g^	3.2 (6.6)	3.2 (7.1)	3.2 (6.4)	*t* = − .068, df = 281	0.946
ALS-SF—total	1.2 (0.71)	1.1 (0.65)	1.3 (0.73)	*t* = − 1.890, df = 291	0.060
ALS-SF anxiety-depression	1.4 (0.87)	1.3 (0.83)	1.5 (0.90)	*t* = − 1.168, df = 291	0.244
ALS-SF depression-elation	1.4 (0.74)	1.3 (0.71)	1.4 (0.76)	*t* = − 1.453, df = 291	0.147
ALS-SF anger	0.80 (0.78)	0.67 (0.75)	0.90 (0.80)	*t* = − 2.559, df = 291	**0.011**

*SFS* Social Functioning Scale, *WASI* Wechsler Abbreviated Scale of Intelligence, *PAS* Premorbid Adjustment Scale, *PANSS* Positive and Negative Syndrome Scale, *YMRS* Young Mania Rating Scale, *AUDIT* The Alcohol Use Disorders Identification Test, *DUDIT* Drug Use Disorders Identification Test, *ALS*-*SF* Affective Lability Scale Short Form

Statistically significant *p* values are in bold

^a^99% (*n* = 290) participants had data on duration of illness

^b^93% (*n* = 273) participants had data on IQ

^c^78% (*n* = 228) had data on duration of untreated illness

^d^98.2% (*n* = 288) participants had data on PAS

^e^99% (*n* = 290) participants had data on YMRS

^f^96.9% (*n* = 284) participants had data on AUDIT

^g^96.6% (*n* = 283) participants had data on DUDIT

### Bivariate analyses in the total sample

Overall, although correlation coefficients are low to moderate, the analyses revealed significant associations between all of the ALS-SF subdimension scores and the SFS interpersonal score (anxiety-depression *p* < 0.001, depression-elation *p* = 0.003, anger *p* < 0.001), as well as the total score (*p* < 0.001). The SFS interpersonal score was further significantly associated with current manic symptoms, current positive and negative psychotic symptoms, current anxiety and depressive symptoms, duration of untreated illness, total number of illness episodes, as well as premorbid social functioning in childhood (see Table 2 for correlation coefficients). The SFS interpersonal score was not associated with sex, age, illness onset at or before 18, duration of illness, IQ, alcohol- or drug misuse, or the psychosis lifetime variable.

**Table 2:** Bivariate correlation analyses

	Total sample	Schizophrenia-spectrum	Bipolar-spectrum
	SFS interpersonal	SFS interpersonal	SFS interpersonal
Sex	*r*_s_ = 0.032	*r*_s_ = 0.152	*r*_s_ = − 0.126
Age	*r*_s_ = 0.020	*r*_s_ = − 0.031	*r*_s_ = − 0.016
IQ	*r* = 0.117	*r* = − 0.003	*r* = 0.133
PANSS P	*r*_s_ = − 0.391**	*r*_s_ = − 0.426**	*r*_s_ = − 0.203**
PANSS N	*r*_s_ = − 0.399**	*r*_s_ = − 0.470**	*r*_s_ = − 0.236**
PANSS G2	*r*_s_ = − 0.236**	*r*_s_ = − 0.295**	*r*_s_ = − 0.236**
PANSS G6	*r*_s_ = − 0.174**	*r*_s_ = − 0.212*	*r*_s_ = − 0.208**
YMRS	*r*_s_ = − 0.142**	*r*_s_ = − 0.161	*r*_s_ = − 0.111
AUDIT	*r* = 0.092	*r* = 0.103	*r* = 0.011
DUDIT	*r*_s_ = − 0.041	*r*_s_ = − 0.013	*r*_s_ = 0.019
Psychosis lifetime	*r*_s_ = − 0.052	n0.a	*r*_s_ = 0.092
Premorbid social functioning	*r*_s_ = − 0.218**	*r*_s_ = − 0.308**	*r*_s_ = − 0.125
Duration of untreated illness	*r*_s_ = − 0.197**	*r*_s_ = − 0.229*	*r*_s_ = − 0.051
Total number of illness episodes	*r*_s_ = 0.175**	*r*_s_ = 0.098	*r*_s_ = 0.046
Duration of illness	*r*_s_ = 0.034	*r*_s_ = − 0.076	*r*_s_ = − 0.035
ALS-SF total	*r* = − 0.244**	*r* = − 0.240**	*r* = − 0.303**
ALS-SF anxiety-depression	*r* = − 0.283**	*r* = − 0.308**	*r* = − 0.304**
ALS-SF depression-elation	*r* = − 0.171**	*r* = − 0.103	*r* = − 0.265**
ALS-SF anger	*r* = − 0.208**	*r* = − 0.246**	*r* = − 0.249**

*SFS* Social Functional Scale, *PANSS* *P* Positive and Negative Syndrome Scale Positive subscale, *PANSS* *N* Positive and Negative Syndrome Scale Negative subscale, *PANSS* *G2* Positive and Negative Syndrome Scale anxiety item, *PANSS* *G6* Positive and Negative Syndrome Scale depression item, *YMRS* Young Mania Rating Scale, *AUDIT* The Alcohol Use Disorders Identification Test, *DUDIT* The Drug Use Disorders Identification Test, ALS-SF Affective Lability Scale Short Form, *p < 0.05, **p < 0.01

### Results from multivariate analyses in the total sample

After controlling for potential confounders, higher scores on the anxiety-depression dimension of the ALS-SF were significantly and independently associated with lower social functioning (*p* = 0.019; model *F* = 8.249, df = 11, *p* < 0.001). In addition, higher levels of current positive- and negative symptoms and lower premorbid social adjustment were significantly associated with lower social functioning (*R*^2^ for the final model = 0.298; PANSS N p < 0.001; PANSS P *p* = 0.008; PAS-S *p* = 0.009, see Table 3). The depression-elation and anger dimensions of the ALS-SF were not significantly associated with social functioning after controlling for potential confounders (*p* = 0.306 and *p* = 0.627, respectively). The *R*^2^ change for the ALS-SF dimensions block was 3.1%, which is a statistically significant contribution (Sig. F change 0.027). The *R*^2^ change for the first block with individual characteristics (PAS-S) was 4.2%, the illness course variables block was 1.9%, and the current symptoms block was 20.6%.

**Table 3:** Multiple linear regression analysis on the relationship between social functioning and affective lability in the total sample

Covariates	Beta	*t* test	*p* value	95% CI for B	
				Lower bound	Upper bound
Premorbid social functioning	− 0.156	− 2.656	**0.009**	− 3.034	− 0.449
Duration of untreated illness	0.004	0.064	0.949	− 1.057	1.128
Total number of illness episodes	0.034	0.553	0.581	− 0.140	0.249
Anxiety (PANSS G2)	− 0.068	− 0.959	0.339	− 4.101	1.417
Depression (PANSS G6)	− 0.051	− 0.746	0.456	− 3.618	1.631
PANSS P	− 0.211	− 2.685	**0.008**	− 2.431	− 0.373
PANSS N	− 0.249	− 3.650	**0.000**	− 2.072	− 0.619
YMRS	0.001	0.016	0.987	− 0.924	0.938
ALS-SF anxiety-depression	− 0.229	− 2.357	**0.019**	− 12.487	− 1.113
ALS-SF depression-elation	0.091	1.027	0.306	− 2.922	9.274
ALS-SF anger	− 0.039	− 0.486	0.627	− 6.610	3.995

*PANSS* *G2* Positive and Negative Syndrome Scale anxiety item, *PANSS* *G6* Positive and Negative Syndrome Scale depression item, *YMRS* Young Mania Rating Scale, *PANSS* *P* Positive and Negative Syndrome Scale Positive subscale, *PANSS* *N* Positive and Negative Syndrome Scale Negative subscale *ALS*-*SF* Affective Lability Scale Short Form

Statistically significant *p* values are in bold

### Follow-up bivariate analyses in diagnostic subgroups

Overall, elevated affective lability was significantly and negatively associated with social functioning in both the schizophrenia- and the bipolar spectrum groups (ALS-SF total score *p* < 0.01, see Table 2). With respect to the ALS-SF subdimensions, the anxiety-depression and the anger dimensions were significantly associated with the SFS in the schizophrenia spectrum group (*p* = 0.001 and *p* = 0.006, respectively). The SFS was further significantly associated with current positive and negative psychotic symptoms, current anxiety and depressive symptoms, premorbid social adjustment in childhood and duration of untreated illness in this group. In the bipolar spectrum group, the association between affective lability and social functioning was significant for all subdimensions (*p* ≤ 0.001). Here, current anxiety and depressive symptoms and positive and negative psychotic symptoms were also significantly associated with the SFS.

### Results from follow-up multivariate analyses in diagnostic subgroups

In the schizophrenia spectrum group, lower social functioning was significantly associated with lower premorbid social functioning in childhood and higher levels of current negative symptoms (*p* < 0.001 and *p* = 0.001, respectively; model *F* = 9.281, df = 7, *p* < 0.001, *R*^2^ for the final model = 0.394), in addition to trend level associations for higher levels of current positive psychotic- (*p* = 0.055) and depressive symptoms (*p* = 0.053). Affective lability (ALS-SF total score) was no longer significantly associated with social functioning after correcting for the level of positive symptoms (*p* = 0.685). There was also a statistically significant association between the ALS total score and the level of positive psychotic symptoms. The analysis thus indicated that the effect of the ALS on the SFS score was mediated through positive psychotic symptoms. In the bipolar spectrum group, elevated affective lability was the strongest predictor of reduced social functioning (*p* = 0.004; model *F* = 6.432, df = 5, *p* < 0.001; *R*^2^ for the final model = 0.164). In addition, a higher level of current positive psychotic symptoms was also significantly associated with reduced social functioning in the bipolar spectrum group (*p* = 0.031). Please refer to supplementary information for regression tables for the subgroups.

## Discussion

### Affective lability and social functioning in the total sample

In the current study, we found that higher scores on the anxiety-depression dimension of the ALS-SF were significantly associated with lower social functioning in severe mental disorders. Albeit accounting for a modest part of the total variance, this association remained at a level of statistical significance even when we controlled for other well-established predictors of social functioning such as premorbid social functioning, duration of untreated illness, and level of current symptoms. We have previously found that affective lability in our sample was characterized by fluctuations between both anxiety-depression and depression-elation across diagnostic groups [22]. However, only fluctuations between anxiety- and depressive symptoms appear to be directly linked to social functioning. As a majority of the sample (58%) consisted of individuals with bipolar disorder, it was somewhat surprising that an association between the depression-elation dimension and social functioning was not found. This might be an indication that internalizing thoughts and behaviors related to negative affectivity are more disrupting to social functioning compared to externalizing problems that may arise as a result of fluctuations in elation.

In line with some previous studies [25, 26], higher levels of current psychotic symptoms (both positive and negative) contributed the most to reduced social functioning in the total sample, highlighting the importance of achieving symptom remission. From an illness course perspective, whether the participants had previous psychotic episodes or not in their lifetime did not appear to influence the level of social functioning. In an earlier study, we found that elevated affective lability was associated with higher levels of positive psychotic symptoms in schizophrenia spectrum disorders, although directionality could not be inferred [21]. Based on the past and current findings, one may, however, speculate that targeting affective lability in treatment might be beneficial for social functioning in psychotic disorders directly but also via reducing positive psychotic symptoms. Interestingly, while affective symptoms (depressive and manic) were associated with social functioning in the bivariate analyses, their statistical significance was not upheld when entered together with affective lability into the multivariate regression model. We tentatively interpret this as support for the claim that affective lability in the anxiety-depression dimension is indeed a “trait-like” illness feature associated with social functioning independent of elevation in symptom levels.

### Affective lability and social functioning in diagnostic subgroups

The follow-up analyses in diagnostic subgroups showed that the significant association between affective lability and social functioning was lost in the schizophrenia spectrum group when other predictors of social functioning were entered into the regression model. Further analyses indicated that the effect of affective lability on social functioning was largely mediated through positive psychotic symptoms in the schizophrenia spectrum group. As noted above, we have also previously reported a significant association between elevated affective lability and increased positive psychotic symptoms in schizophrenia spectrum disorders [21]. Since elevated affective lability is considered a more stable trait that may increase the risk for reality distortion, in line with the notion of an affective pathway to psychosis [68], we interpret our findings as mediation. However, the cross-sectional study design does not rule out the possibility that high levels of positive psychotic symptoms are followed by higher affective lability. More studies, preferably using longitudinal designs, are needed to clarify these relationships in schizophrenia spectrum disorders. In the bipolar spectrum group, on the other hand, affective lability remained significantly and independently associated with social functioning even when the other predictors were taken into account. Nonetheless, as our previous study showed that the level of affective lability is significantly different in BDI versus BDII disorders [22], this finding warrants further investigation in larger samples to tease out if the association between affective lability and social functioning is the same irrespective of bipolar subtype.

### Putative mechanisms underlying the relationship between affective lability and social functioning

Healthy social relationships are tied to longer, healthier lives and improved psychological well-being [69]. Thus, improving social functioning should be an important treatment goal in all psychiatric disorders. In fact, research indicates that social factors such as social support and social integration are at least as important for mortality as well-established behavioral risk factors such as smoking, obesity, physical inactivity and high blood-pressure [70]. In severe mental disorders, where life expectancy has been found to be substantially decreased compared to the general population, the health-promoting effects of social factors are perhaps particularly crucial [68–70]. The results of the current study indicate that elevated affective lability may be an obstacle to harvesting the benefits of social interactions, although the directionality and the exact mechanisms by which this may exert its effects are, thus far, unclear.

Negative affect has been found to predict social functioning across schizophrenia and bipolar disorder, and high levels of negative affect have been linked to greater fluctuations in affective states [10, 71]. This has again been associated with delayed return to a more adaptive affective baseline which can result in adverse health effects [72–74]. One can speculate that a pattern with elevated affective lability, high levels of negative affect and slow return to a neutral physiological state could give rise to a vicious cycle, fostering coping behaviors that are counterproductive to social functioning, such as withdrawal, avoidance and disengagement. Over time, this may interfere with the drive to forge and maintain both peripheral and close social connections [75–78], which is deleterious to well-being and longevity [79, 80]. Feeding into this potential negative cycle, social settings are in themselves triggering to a host of different affective experiences due to their ever-changing, ambiguous and unpredictable nature. Successful social navigation is therefore contingent upon having a clear representation of one’s own internal affective state to guide appropriate behavior and responses [81]. Elevated affective lability might make it distinctively more difficult to differentiate, categorize and label affective states in a precise and specific way, i.e. result in low emotional granularity [82], which has further been associated with social dysfunction [83–86]. Collectively and tentatively, affective lability may contribute to steering individuals away from the social world while features in the social world, in turn, may increase affective lability, generating negative interactions that contribute to impairments in social functioning.

### Limitations and strengths

The findings of the present study must be interpreted in light of some limitations. Causal attributions are precluded due to the cross-sectional nature of the study and data on comorbid disorders such as personality disorders, ADHD and anxiety disorders are lacking. In addition, since this is a naturalistic study, the participants have typically received a broad range, as well as different combinations, of pharmacological and psychosocial treatments that would be very difficult to control for in the statistical analyses. Furthermore, as the ALS-SF and the SFS are based on self-report, there is a risk for recall- and response bias that cannot be ruled out. Finally, the measures used for anxiety and depressive symptoms are based on a scale primarily developed for assessing psychotic symptoms. Hence, the possibility that current symptoms still could have influenced the association between affective lability and social functioning cannot be ruled out completely. However, we believe that the likelihood of this is limited due to the relatively low levels of anxiety and depressive symptoms. The study also has several strengths; it demonstrates that there are associations between affective lability and social functioning in a large, diagnostically well-characterized sample of participants with severe mental disorders while accounting for many other well-documented confounding variables. To our knowledge, this has not been shown previously.

## Conclusions

Our results indicate that elevated affective lability may have a negative impact on social functioning in severe mental disorders. If replicated, this could have important clinical implications as affective lability can be targeted in treatment through various forms of emotion regulation skills training. Future research should address whether a therapeutic focus on affective lability could be one pathway towards improving social outcomes in severe mental disorders.

## Supplementary Information

Below is the link to the electronic supplementary material.

Supplementary file1 (DOCX 14 KB)

## Data Availability

The data that support the findings of this study will be made available upon reasonable request.
