# Supplementary material for: Affective lability and social functioning in severe mental disorders
Source: Eur Arch Psychiatry Clin Neurosci. 2022 Jan 27;272(5):873–85. doi: 10.1007/s00406-022-01380-1 (PMC9279216; doi:10.1007/s00406-022-01380-1)
Supplement: Supplementary file 1 — Supplementary file1 (DOCX 14 KB) [file 406_2022_1380_MOESM1_ESM.docx]

Supplementary 1

Multiple linear regression analysis on the relationship between social functioning and affective lability in the schizophrenia-spectrum group

| Covariates | Beta | t-test | p-value | 95%CI for B |  |
| --- | --- | --- | --- | --- | --- |
|  |  |  |  | Lower bound | Upper bound |
| Premorbid social functioning | -.268 | -3.363 | **.001** | -4.870 | -1.256 |
| Duration of untreated illness | -.022 | -.277 | .782 | -.029 | .022 |
| PANSS P | -.179 | -1.945 | .055 | -2.234 | .022 |
| PANSS N | -.330 | -3.915 | **.000** | -2.394 | -.784 |
| Anxiety (PANSS G2) | -.124 | -1.347 | .181 | -6.891 | 1.319 |
| Depression (PANSS G6) | -.175 | -1.959 | .053 | -7.747 | .049 |
| ALS-SF total | -.035 | -.407 | .685 | -8.771 | 5.786 |
|  |  |  |  |  |  |

*PANSS G2=Positive and Negative Syndrome Scale anxiety item, PANSS G6= Positive and Negative Syndrome Scale depression item, PANSS P= Positive and Negative Syndrome Scale Positive subscale, PANSS N= Positive and Negative Syndrome Scale Negative subscale, ALS-SF=Affective Lability Scale Short Form.*

Multiple linear regression analysis on the relationship between social functioning and affective lability in the bipolar-spectrum group

| Covariates | Beta | t-test | p-value | 95%CI for B |  |
| --- | --- | --- | --- | --- | --- |
|  |  |  |  | Lower bound | Upper bound |
| PANSS P | -.166 | -2.182 | **.031** | -3.031 | 1.151 |
| PANSS N | -.148 | -1.878 | .062 | -2.864 | .072 |
| Depression (PANSS G6) | .035 | .390 | .697 | -2.430 | 3.627 |
| Anxiety (PANSS G2) | -.097 | -1.129 | .261 | -4.630 | 1.261 |
| ALS-SF total | -.239 | -.2.956 | **.004** | -13.049 | -2.598 |
|  |  |  |  |  |  |

*PANSS G2=Positive and Negative Syndrome Scale anxiety item, PANSS G6= Positive and Negative Syndrome Scale depression item, PANSS P= Positive and Negative Syndrome Scale Positive subscale, PANSS N= Positive and Negative Syndrome Scale Negative subscale, ALS-SF=Affective Lability Scale Short Form.*
